# Parafoveal chondral lesion of the femoral head in patients with femoroacetabular impingement

**DOI:** 10.1590/0100-3984.2023.0019

**Published:** 2023

**Authors:** Guilherme Pradi Adam, Igor Varela Meyer, Tainá de Arruda e Silva, Mark Wanderley, Eduardo Bristot de Mello, Leonardo Valentim, Israel Silva Maia, Mariela Goulart Adames

**Affiliations:** 1 Clínica Imagem, Florianópolis, SC, Brazil; 2 Instituto de Ensino e Pesquisa Hospital Baia Sul, Florianópolis, SC, Brazil; 3 Universidade do Sul de Santa Catarina, Palhoça, SC, Brazil

**Keywords:** Arthrography, Cartilage diseases, Femoracetabular impingement, Hip joint, Magnetic resonance imaging, Tomography, X-ray computed, Artrografia, Doenças das cartilagens, Impacto femoroacetabular, Articulação do quadril, Ressonância magnética, Tomografia computadorizada

## Abstract

**Objective:**

To describe cases of parafoveal chondral lesion of the femoral head in patients with femoroacetabular impingement, correlating the clinical and imaging data.

**Materials and Methods:**

This was a retrospective descriptive case series of parafoveal chondral lesion of the femoral head in 21 patients who underwent computed tomography and magnetic resonance arthrography scans of the hip, having then received an imaging-based diagnosis of femoroacetabular impingement.

**Results:**

Of the 21 patients evaluated, 15 (71%) had cam-type femoroacetabular impingement, whereas five (24%) had mixed-type impingement, and one (5%) had pincer-type impingement. Twelve patients (57%) had a low frequency of physical activity, which was significantly associated with the presence of cam-type impingement (*p* = 0.015). Although the extent of the lesion correlated significantly with the acetabular coverage angle (*p* = 0.04), it did not correlate significantly with the alpha angle or femoral head-neck offset value (*p* = 0.08 and *p* = 0.06, respectively). We also found no correlation between the extent of the lesion and the other main parameters that define the femoroacetabular impingement types.

**Conclusion:**

This was one of the largest case series of parafoveal chondral lesion of the femoral head in patients with imaging findings of femoroacetabular impingement. The extent of such lesions does not appear to correlate with the parameters of femoroacetabular impingement, with the exception of the acetabular coverage angle.

## Abstract (pt)

**Objetivo:**

Descrever casos de lesão condral parafoveal da cabeça femoral em pacientes com impacto femoroacetabular, correlacionando dados clínicos e de imagem.

**Materiais e Métodos:**

Esta foi uma série de casos descritiva retrospectiva de lesão condral parafoveal da cabeça femoral em 21 pacientes submetidos a tomografia computadorizada e artrorressonância magnética do quadril e que receberam diagnóstico por imagem de impacto femoroacetabular.

**Resultados:**

Dos 21 pacientes avaliados, 15 (71%) tiveram impacto femoroacetabular do tipo cam, enquanto cinco (24%) tiveram impacto do tipo misto e um (5%) teve impacto do tipo pincer. Doze pacientes (57%) apresentaram baixa frequência de atividade física, sendo esta significativamente associada a impacto do tipo cam (*p* = 0,015). Houve correlação significativa entre a extensão da lesão e o ângulo de cobertura acetabular (*p* = 0,04), porém, não se correlacionou significativamente com o ângulo alfa ou com o valor do deslocamento cabeça-colo femoral (*p* = 0,08 e *p* = 0,06, respectivamente). Também não encontramos correlação entre a extensão da lesão e os outros principais parâmetros que definem os tipos de impacto femoroacetabular.

**Conclusão:**

Esta foi uma das maiores casuísticas de lesão condral parafoveal da cabeça femoral em pacientes com achados de imagem de impacto femoroacetabular. A extensão dessas lesões não parece se correlacionar com os parâmetros do impacto femoroacetabular, com exceção do ângulo de cobertura acetabular.

## INTRODUCTION

Femoroacetabular impingement is a major cause of early osteoarthritis of the hip, especially in young, active patients, usually between 20 and 40 years of age, with an estimated prevalence of 10-15%^(1)^. It is characterized by pathological contact between the bony prominences of the acetabulum and femur during movement of the hip joint; that limits the range of physiological movement, typically of flexion and internal rotation^(1-3)^. During sports, as well as during activities of daily living, repetitive microtraumas occur on the femoroacetabular bone surfaces. As a result, there is damage to the labrum and progressive, irreversible damage to the cartilage, resulting in degenerative disease of the hip joint^(1)^.

Two mechanisms are often described to explain the mechanics of femoroacetabular impingement, corresponding to two types. The first, known as cam, which is most common in young male patients and in athletes, is characterized by a nonspherical femoral head with a prominent head-neck junction. The second type, known as pincer, is most common in middle-aged female patients and is characterized by excessive (diffuse or focal) acetabular coverage^(3)^.

**Figure 1:**
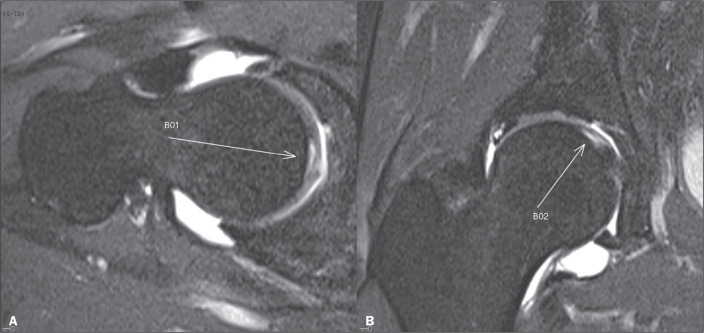
Coronal and axial oblique proton-density fat-saturated MR arthrography of the right hip (A and B, respectively), showing a parafoveal chondral lesion of the femoral head (arrows), with delamination.

Magnetic resonance imaging (MRI) is the standard noninvasive imaging method of choice to evaluate changes in the hip joint (Figure 1), with an estimated sensitivity of 94% and 92% for the detection of labral and chondral lesions, respectively^(4)^. In most cases of femoroacetabular impingement, MRI shows a loss of the intermediate signal of the hyaline cartilage; it can also identify discrete fissures, which appear as lines of high signal intensity crossing the articular cartilage^(5)^.

Previous studies have described the types of chondral and labral injury most commonly associated with femoroacetabular impingement. It has been suggested that repeated microtraumas (due to contact between the femur and acetabulum) result in ruptures at the chondrolabral junction, particularly in the anterior superior labrum, which predispose to cartilage damage in the adjacent joint^(5-7)^. However, the pattern of chondral damage relates to each unique anatomical deformity, as well as to the type of activity performed, therefore varying among patients.

There have been few previous reports describing one specific type of focal injury: that occurring in the parafoveal cartilage region of the femoral head in patients with femoroacetabular impingement^(6)^. The focus of this study was to perform a retrospective analysis of a series of cases of parafoveal chondral lesion of the femoral head in patients with femoroacetabular impingement who underwent MRI, correlating clinical data and imaging findings.

## MATERIALS AND METHODS

This was a retrospective descriptive case series that included patients with an imaging-based diagnosis of femoroacetabular impingement and chondral lesions on the medial face of the femoral head near the borders of the fovea capitis femoris. All of the patients selected had undergone computed tomography (CT) and MR arthrography scans of the hip between 2017 and 2019 at Clínica Imagem, in the city of Florianópolis, Brazil. The type of femoroacetabular impingement and the extent of the parafoveal lesions were determined from imaging examinations. Demographic data were collected from the patient database. The study was approved by the local research ethics committee (Reference no. 28097019.9.0000.0115.115). Because of the retrospective nature of the study, the requirement for informed consent was waived.

The MR arthrography images were acquired in a 1.5-T scanner (Avanto; Siemens Healthcare, Erlangen, Germany), at a slice thickness of 3 mm, in proton density-weighted sequences, with and without fat saturation, in the axial, coronal, and sagittal planes. Fluoroscopy-guided intra-articular injection of contrast medium was performed, as were twoand three-dimensional CT reconstructions in a multidetector CT scanner (Somatom Definition AS 128; Siemens Healthcare). The procedures described were performed after the patient had been informed of the risks, aseptic procedures had been carried out, and gadolinium-based contrast medium (0.3 mL), together with saline solution (20 mL), had been administered by fluoroscopy-guided intra-articular injection. After local anesthesia with 5 mL of 2% lidocaine, 13 mL of the gadolinium-saline solution had been injected without resistance. No complications were reported during or after these procedures.

The imaging examinations were analyzed by two radiologists specializing in musculoskeletal imaging, with three and four years of experience, respectively, working independently. Variations of up to 10% in the measures were considered acceptable, the highest values being registered. If a value recorded by one radiologist differed from that of the other by more than 10% or if there was an inter-rater difference that resulted in a change in the classification of a lesion, a senior radiologist, with 13 years of experience, analyzed the imaging examination to resolve the disagreement.

The categorization of femoroacetabular impingement as cam, pincer, or mixed type was based on the standards described in the literature:

- The alpha angle was measured in the axial oblique plane by using sectional methods. The alpha angle is defined as the intersection between a line drawn along the axis of the femoral neck and another extending from the center of the femoral head to the point where the circumference of the head is intercepted by the border of the femoral neck^(8)^. An alpha angle ≥ 55° was categorized as pathological, in accordance with most of the data in the literature^(9-12)^.
- The femoral head-neck offset was defined as the distance between the anterior margin of the femoral head-neck junction and the anterior margin of the femoral head. It was categorized as pathological if it was < 8 mm^(8-10)^.
- Acetabular coverage was quantified by the Wiberg method (measurement of the lateral center edge angle), by using reconstruction of CT images acquired in the coronal plane. A line was drawn from the center of the femoral head to the outer margin of the acetabulum, intersecting with a vertical line drawn from the center of the femoral head, perpendicular to the horizontal line that passes between the ischial tuberosities. An acetabular angle ≥ 40° was considered indicative of coxa profunda or excessive total acetabular coverage^(8-10)^.
- Acetabular version was measured on axial CT images by drawing a line between the anterior and posterior edge of the acetabulum and another, vertical, line from the posterior edge, tangential to a horizontal line connecting the posterior edges of the acetabulum. It was considered normal when in anteversion. When in retroversion (< 15°), it was considered suggestive of the pincer type of impingement^(8-10)^.

The severity of each chondral lesion was classified by consensus between the two radiologists, on the basis of the International Cartilage Repair Society classification^(13,14)^. Parafoveal chondral lesions of the femoral head were considered, by definition, chondral lesions on the medial face of the femoral head in the vicinity of the fovea capitis femoris. Chondral lesions that extended to the rest of the femoral head or that were caused by end-stage osteoarthritis of the hip were excluded.

The results were entered into a Microsoft Excel spreadsheet and exported for analysis to the Stata statistical software package, version 15.0 (StataCorp LP, College Station, TX, USA). Quantitative variables are expressed as median and interquartile range, the categorical variables being compared by using Fisher’s exact test. Linear regression was performed to quantify the associations among the extent of the parafoveal chondral lesion of the femoral head, the alpha angle, and the degree of femoral head-neck offset, given that 95% of the patients in our sample had the cam or mixed type of impingement. Values of *p* < 0.05 were considered significant, without any adjustment for multiple comparisons. To determine how well the extent of the lesion correlated with the main parameters that define the types of femoroacetabular impingement, Spearman’s correlation coefficient was used.

## RESULTS

We evaluated the cases of 21 patients with femoroacetabular impingement and parafoveal chondral lesion of the femoral head, evaluated between 2017 and 2019. There were no cases of parafoveal chondral lesion of the femoral head without imaging findings of femoroacetabular impingement. The demographic data were analyzed for all 21 patients. Because some data were missing, seven patients were excluded from the analyses of origin, history of hip surgery, comorbidities, and frequency of physical activity. Of the remaining 14 patients, eight (57%) had a low frequency of physical activity, which was associated with the presence of cam-type impingement (*p* = 0.015). The main demographic data are presented in Table 1.

**Table 1:** Demographic and clinical characteristics of the participants.

Characteristics	(N = 21)
Age (years), median (IQR)	42 (36-52)
Gender, n (%)	
Female	11 (52)
Male	10 (48)
Patient origin, n (%)^*^	
Florianópolis	11 (79)
Other	3 (21)
History of hip surgery, n (%)^*^	
Yes	2 (14)
No	12 (86)
Comorbidities, n (%)^*^	
Yes	1 (7)
No	13 (93)
Frequency of physical activity, n (%)^*^	
< 3 times a week	8 (57)
≥ 3 times a week	6 (43)

IQR, interquartile range.

* Data available for only 14 patients.

Among the 21 patients evaluated, the femoroacetabular impingement was of the cam type in 15 (71%), the mixed type in five (24%), and the pincer type in one (5%), as shown in Table 2. It was observed that for every 1-degree increase in the alpha angle, there was an increase of 0.24 mm in the diameter of the lesion, adjusted for the femoral head-neck offset (95% CI: 0.04 to 0.52). In addition, for every 1.00-mm increase in the femoral head-neck offset, there was an increase of 1.08 mm in the size of the lesion, adjusted for the alpha angle (95% CI: -0.15 to 2.3). However, neither of those correlations was statistically significant (*p* = 0.09 and *p* = 0.08, respectively). There was a significant correlation between the extent of the lesion and the acetabular coverage angle (*p* = 0.04), There was no significant correlation between the extent of the lesion and the alpha angle or femoral head-neck offset value (*p* = 0.08 and *p* = 0.06, respectively).

**Table 2:** Imaging characteristics of the participants.

Variable	(N = 21)
Chondropathy grade	
3a	8 (38)
3b	10 (48)
3c	2 (9)
4a	1 (5)
Type of femoroacetabular impingement	
Cam	15 (71)
Pincer	1 (5)
Mixed	5 (24)
Parafoveal chondral lesion size (mm), median (IQR)	9.0 (6.0-13.0)
Head-neck offset (mm), median (IQR)	3.1 (2.0-4.4)
Femoral neck angle (°), median (IQR)	132 (131-133)
Acetabular version (°), median (IQR)	22 (13-25)
Acetabular coverage (°), median (IQR)	30 (24-35)
Alpha angle (°), median (IQR)	60 (56-64)

IQR, interquartile range.

## DISCUSSION

This is one of the few reports of parafoveal chondral lesion of the femoral head and its association with femoroacetabular impingement. In our sample, there was a predominance of middle-aged female patients, a low frequency of physical activity, and a high prevalence of the cam-type impingement morphology. As previously stated, the extent of the parafoveal lesion was not found to correlate with the alpha angle or femoral head-neck offset value, although it did correlate significantly with the acetabular coverage angle.

The predominance of cam-type femoroacetabular impingement (71%) in our case series is in accordance with the findings of Zaltz et al.^(6)^ in a sample of patients with similar cartilaginous lesions. It has been suggested that cam-like bone deformity is associated with varying degrees of damage to the peripheral articular cartilage and ruptures at the chondrolabral junction, secondary to repeated microtraumas resulting from contact between the femur and acetabulum^(6,15,16)^. The predominance of females is inconsistent with the findings of prior studies, which reported the prevalence of cam-type impingement to be greater among males^(17-19)^, although the difference between the sexes is poorly understood. That discrepancy could be attributable to the small size of our sample and the fact that it was a convenience sample, as well as to the fact that we selected only patients with femoroacetabular impingement who also had at least one parafoveal chondral lesion of the femoral head.

To our knowledge, this is the first study to describe a large, non-athlete population of patients with parafoveal chondral lesions of the femoral head who have undergone CT and MR arthrography scans of the hip. It is also, to our knowledge, the first to report the absence of a correlation between the extent of the parafoveal chondral lesion of the femoral head and most of the angles and measures that define femoroacetabular impingement, with the exception of the acetabular coverage angle. We believe that there must be another pathophysiological mechanism involved in the development of such lesions, unlike lesions of the anterosuperior margin of the acetabulum, which are more related to the cam-type impingement morphology.

Our study has some limitations. First, the sample size was small, which could have resulted in a selection bias. In addition, the retrospective study design could have limited the reliability of the information collected. Furthermore, there was no control group of patients with similar chondral lesions without femoroacetabular impingement. Other limitations include the lack of correlation with hip arthroscopy findings and the fact that the diagnosis of femoroacetabular impingement was based solely on imaging criteria, which were not correlated with clinical data or physical examination findings.

## CONCLUSIONS

We have described one of the largest case series of parafoveal chondral lesion of the femoral head in patients with femoroacetabular impingement. The extent of the chondral lesion does not appear to correlate with most of the parameters of femoroacetabular impingement, the one exception being the acetabular coverage angle.
